# Activated AKT/PKB signaling in *C. elegans *uncouples temporally distinct outputs of DAF-2/insulin-like signaling

**DOI:** 10.1186/1471-213X-6-45

**Published:** 2006-10-04

**Authors:** Minaxi S Gami, Wendy B Iser, Keaton B Hanselman, Catherine A Wolkow

**Affiliations:** 1 Laboratory of Neurosciences, NIA, NIH, Baltimore, MD, USA

## Abstract

**Background:**

In the nematode, *Caenorhabditis elegans*, a conserved insulin-like signaling pathway controls larval development, stress resistance and adult lifespan. AGE-1, a homolog of the p110 catalytic subunit of phosphoinositide 3-kinases (PI3K) comprises the major known effector pathway downstream of the insulin receptor, DAF-2. Phospholipid products of AGE-1/PI3K activate AKT/PKB kinase signaling via PDK-1. AKT/PKB signaling antagonizes nuclear translocation of the DAF-16/FOXO transcription factor. Reduced AGE-1/PI3K signaling permits DAF-16 to direct dauer larval arrest and promote long lifespan in adult animals. In order to study the downstream effectors of AGE-1/PI3K signaling in *C. elegans*, we conducted a genetic screen for mutations that suppress the constitutive dauer arrest phenotype of *age-1(mg109) *animals.

**Results:**

This report describes mutations recovered in a screen for suppressors of the constitutive dauer arrest (daf-C) phenotype of *age-1(mg109)*. Two mutations corresponded to alleles of *daf-16*. Two mutations were gain-of-function alleles in the genes, *akt-1 *and *pdk-1*, encoding phosphoinositide-dependent serine/threonine kinases. A fifth mutation, *mg227*, located on chromosome X, did not correspond to any known dauer genes, suggesting that *mg227 *may represent a new component of the insulin pathway. Genetic epistasis analysis by RNAi showed that reproductive development in *age-1(mg109);akt-1(mg247) *animals was dependent on the presence of *pdk-1*. Similarly, reproductive development in *age-1(mg109);pdk-1(mg261) *animals was dependent on *akt-1*. However, reproductive development in *age-1(mg109); mg227 *animals required only *akt-1*, and *pdk-1 *activity was dispensable in this background. Interestingly, while *mg227 *suppressed dauer arrest in *age-1(mg109) *animals, it enhanced the long lifespan phenotype. In contrast, *akt-1(mg247) *and *pdk-1(mg261) *did not affect lifespan or stress resistance, while both *daf-16 *alleles fully suppressed these phenotypes.

**Conclusion:**

A screen for suppressors of PI3K mutant phenotypes identified activating mutations in two known pathway components, providing insights into their regulation. In particular, the interdependence of *akt-1 *and *pdk-1*, even in activated forms, supports the existence of AGE-1-independent pathways for these phospholipid-dependent kinases. Phenotypic analysis of these alleles shows that the larval and adult outputs of AGE-1/PI3K are fully separable in these mutants.

## Background

In the nematode *C. elegans*, development, lifespan and stress resistance are regulated by a conserved insulin-like signaling pathway [1]. Under optimal conditions, *C. elegans *nematodes develop through four larval stages (L1-L4) to become reproductive adults. In adverse environmental conditions, such as low food abundance or high population density, larvae can enter diapause and arrest development as dauer larvae. Dauer larvae are non-feeding, long-lived, sexually immature and stress resistant animals. Morphologically they are thinner and darker than reproductive adults [2,3]. Once favorable conditions resume, dauer larvae can re-enter the reproductive developmental pathway to become fertile adults.

In *C. elegans*, three genetic pathways influence the decision to arrest development at the dauer larval stage [3,4]. One is a neuronal *daf-11 *guanyly lcyclase pathway [4,5]. The second is a TGF-β pathway that regulates dauer arrest through expression of the DAF-7/TGF-β ligand [6]. Ultimately, the *daf-11 *and *daf-7 *pathways act on a nuclear hormone receptor, encoded by *daf-12 *[4,7]. The third pathway regulating dauer arrest is an insulin-like signaling pathway that includes the genes *daf-2*, encoding an insulin/IGF-I receptor-like protein, *age-1*, encoding the p110 catalytic subunit of PI3K, as well as a collection of protein kinases, encoded by the genes *akt-1, -2, pdk-1 *and *sgk-1*, which mediate phospholipid signaling downstream from AGE-1 [8-12]. Phosphoinositide bound PDK-1 activates the protein kinases which, in turn, phosphorylate and inhibit nuclear translocation of the FOXO transcription factor DAF-16 [13-16]. AGE-1/PI3K-generated phospholipids are believed to bind to both PDK-1 and AKT-1. Phosphoinositide bound AKT-1 undergoes a conformational change that promotes phosphorylation by PDK-1 and this phosphorylation is greatly enhanced if PDK-1 is also bound to phosphoinositides. However, kinase activity can also be detected in the absence of phosphoinositides suggesting that phospholipid-binding may not be absolutely required for PDK-1 catalytic activity [17,18].

In addition to regulating dauer arrest in larvae, DAF-2/insulin signaling also regulates lifespan and stress resistance in adult animals [9,19-23]. As with dauer larval arrest, *daf-16 *activity is required for the longevity and stress resistance phenotypes in *daf-2 *pathway mutants [20,21]. DAF-2/insulin signaling regulates the expression of many genes involved in metabolism and stress resistance, including some genes that appear to be direct transcriptional targets of DAF-16, such as *daf-15*/RAPTOR and *sod-3*/Mn-SOD [24-26].

Most of the known *daf-2 *pathway components were identified in genetic screens on the basis of dauer arrest phenotype. In particular, a screen for suppressors of dauer larval arrest in animals with the null *age-1(mg44) *mutation uncovered hypermorphic alleles of *akt-1 *and *pdk-1 *[11,12]. In order to determine whether additional effectors of AGE-1/PI3K signaling could be recovered by this approach, we repeated this screen, using the missense *age-1(mg109) *allele [10].

Here, we describe five mutations identified in this screen, comprising two *daf-16 *alleles and new hypermorphic alleles of *akt-1 *and *pdk-1 *that appear to promote activation of AKT/PKB signaling in the absence of AGE-1/PI3K. The *akt-1 *and *pdk-1 *alleles also uncoupled the larval dauer arrest, adult longevity and stress resistance phenotypes of *age-1(mg109) *animals. We also describe an uncloned allele that has the unusual phenotype of enhancing the longevity of *age-1(mg109) *adult animals. These findings suggest that the larval and adult phenotypes of insulin signaling in *C. elegans *may be differentially affected by AKT/PKB signaling.

## Results and discussion

### Genetic identification of *age-1(mg109) *suppressors

In *C. elegans*, both lifespan and dauer formation are regulated by *age-1 *activity [10,27]. Animals with maternal, but not zygotic, *age-1 *activity do not arrest as dauer larvae, but grow into reproductive adults that are stress resistant and long-lived. In the absence of both maternal and zygotic *age-1 *activity, animals arrest development as dauer larvae. In order to identify genetic modifiers of *age-1*, we conducted a screen for mutations that could suppress the dauer-constitutive (daf-C) phenotype of *age-1(mg109)*, which carries a missense mutation in the lipid kinase domain of AGE-1/PI3K [10]. Previously, a similar genetic screen identified the AKT-1 and PDK-1 effectors of AGE-1/PI3K [11,12]. We repeated this screen in an effort to identify additional mutations capable of activating signaling in the absence of AGE-1/PI3K. From a screen of approximately 20,000 haploid genomes, we identified 40 alleles that could suppress the dauer-constitutive phenotype of *age-1(mg109) *animals (see Methods). Five of these alleles were chosen for further study, based on high penetrance and strong suppression of *age-1(mg109) *constitutive dauer arrest. In particular, four of these alleles, *mg242, mg255, mg261 *and *mg227*, strongly suppressed the *age-1(mg109) *dauer-constitutive phenotype (Table 1). The *mg247 *allele partially suppressed this larval phenotype, as *age-1(mg109);mg247 *animals bypassed dauer arrest but then developed into sterile adults (Table 1). Previous studies have shown that adult sterility is correlated with modest decrements in AGE-1/PI3K signaling [27,28]. In addition to the adult sterility phenotype, *mg247 *animals also exhibited a slight developmental delay, which has also been previously observed in insulin pathway mutants [19,27,28]. Thus, we conclude that the *mg247 *allele only partially restores phospholipid signaling in *age-1(mg109) *animals.

**Table 1:** Suppression of the *age-1(mg109) *dauer arrest phenotype.

Genotype	20°C (% of population)			25°C (% of population)			*(n)*
			
	Dauer larvae	Adults		Dauer larvae	Adults		
					
		Sterile	Fertile		Sterile	Fertile	
Wildtype	0	0	100	0	0	100	(127,109)
*age-1(mg109)*§	100	0	0	100	0	0	(45, 56)
*age-1(mg109);mg242*	0	0	100	0	0	100	(79, 37)
*age-1(mg109);mg255*	0	0	100	0	0	100	(41, 41)
*age-1(mg109);mg247*	0	36.5	64.4	0	25	75*	(71, 25)
*age-1(mg109);mg261*	0	0	100**	0	0	100***	(43, 59)
*age-1(mg109);mg227*	0	0	100 ^#^	0	0	100 ^#^	(65,89)

Some animals exhibited Egl (egg retention) phenotype: * 35 %, ** 4.7 %, *** 32 %, # 100 %.

§ These are homozygote progeny of *age-1(mg109) *homozygous hermaphrodites that have been maternally rescued for dauer arrest [27].

**Figure 1:**
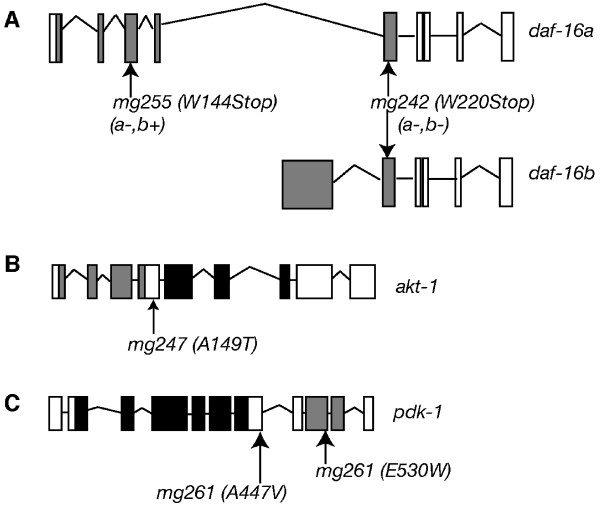
**Mutations identified as suppressors of constitutive dauer arrest in *age-1(mg109) *animals**. Suppressors were mapped using standard SNP-snip-mapping protocols with the Hawaiian strain CB4856 [29]. Mutations were confirmed using PCR and direct DNA sequencing. (A) *daf-16(mg242) *is a Trp220Amber substitution in exon 5, affecting both *daf-16a *and *daf-16b *splice forms. *daf-16(mg255) *is a Trp144Amber substitution in exon 3 that only affects the *daf-16a *splice form. Both mutations fall within the Forkhead domain (indicated by the grey area) involved in DNA interaction. (B) *akt-1(mg247) *is an Ala149Thr substitution that falls in the linker region, between the pleckstrin homology domain (PH, indicated by the grey boxes) and the protein kinase C domain (KD, indicated by the black boxes). (C) *pdk-1(mg261) *is a Ala447Val substitution in the linker region and a Glu530Trp substitution in the pleckstrin homology domain (PH, grey boxes) of PDK-1.

Using standard SNP snip-mapping protocols, we mapped four of these alleles to genetic regions that contained known components of the *age-1 *pathway [29]. Molecular lesions in candidate genes were identified by direct DNA sequencing (see Methods). Two alleles, *mg242 *and *mg255*, corresponded to nonsense mutations in *daf-16 *(Figure 1A). *daf-16(mg242) *is a Trp220Amb nonsense mutation in the forkhead DNA binding domain of DAF-16 and truncates both major *daf-16 *splice forms, *daf-16a *and *daf-16b*, resulting in a predicted null phenotype. *daf-16(mg255) *carries a Trp144Amb nonsense mutation in the forkhead domain of the *daf-16a *splice form only (Figure 1A). Two other suppressor alleles corresponded to mutations in the serine/threonine kinases that are regulated by phosphoinositide (PIP) products of AGE-1/PI3K. The *mg247 *allele corresponded to a missense mutation in *akt-1 *(Ala149Thr). This mutation changes a nonconserved residue in the linker region between the pleckstrin homology (PH) and kinase domains of AKT-1 and is predicted to affect both *akt-1a *and *akt-1b *splice forms (Figure 1B). The *mg261 *allele is the result of two mutations in different regions of PDK-1 of which both are missense mutations. The first corresponds to a nonconserved residue in the linker region between the kinase domain and the PH domain (Ala447Val) and the second in a conserved residue in the PH domain (Glu530Trp) (Figure 1C). A fifth mutation, *mg227*, was mapped to a region of chromosome X that does not contain any known components of the *daf-2 *pathway. Two known dauer pathway genes, *sgk-1 *(X 23.70) and *daf-12 *(X 2.39), are also on chromosome X but are on the outside of the candidate region and thus do not correspond to the gene mutated by *mg227 *(see Methods) [7,8].

**Figure 2:**
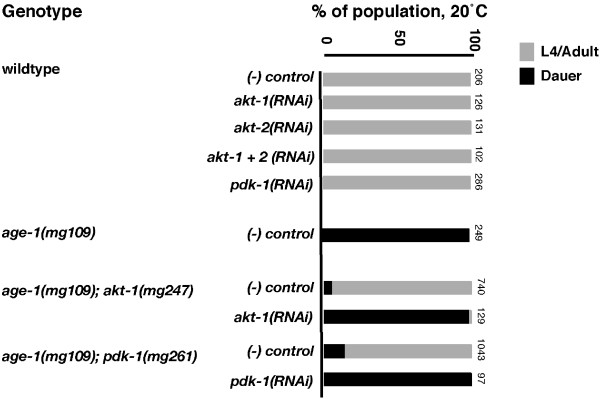
**RNAi against components of the insulin signaling pathway reverts *age-1(mg109) *dauer arrest suppression by *akt-1(mg247) *and *pdk-1(mg261) *mutant alleles**. Animals were fed bacteria containing either control vector, L4440 or vectors producing gene-specific dsRNA. Numbers on the side of the bars represent the total number of animals examined. The bars show the percentage of animals that developed into L4 larvae or adults (grey) and those that arrested as dauers (black). RNAi experiments were diluted with control L4440 RNAi to match the mixed *akt-1 *and *akt-2 *RNAi dose. *akt-1, akt-2 *and *pdk-1 *RNAi experiments were conducted on wildtype, *age-1(mg109), age-1(mg109);akt-1(mg247) *and *age-1(mg109);pdk-1(mg261) *animals

Both *akt-1 *and *pdk-1 *encode proteins that are effectors of signaling downstream of AGE-1/PI3K [11,12]. The observation that the *akt-1(mg247) *and *pdk-1(mg261) *alleles suppressed the daf-C phenotype of *age-1(mg109) *animals suggested these alleles may activate AKT-1 and PDK-1 function in the absence of the phospholipid products of AGE-1 (Table 1). Consistent with this hypothesis, both *akt-1(mg247) *and *pdk-1(mg261) *segregate in a dominant fashion (progeny of heterozygous mothers were scored, *akt-1(mg247)*, 72.2 % bypassers, 27.8 % dauers, n = 467 animals: *pdk-1(mg261)*, 75.8 % bypassers, 24 % dauers, n = 153 animals). To confirm that *mg247 *and *mg261 *were activating mutations, we tested whether RNA interference (RNAi)-mediated expression of these genes could revert their suppression of *age-1(mg109) *dauer arrest. Since *age-1(mg109);akt-1(mg247) *adults were mostly sterile at 25°C, we conducted these experiments at 20°C using the feeding RNAi protocol [30]. Consistent with *mg247 *being a hypermorphic *akt-1 *allele, RNAi of *akt-1 *in *age-1(mg109);akt-1(mg247) *animals fully reversed the suppression of dauer arrest and animals arrested as dauer larvae (Figure 2). Similarly, *pdk-1 *RNAi reversed the dauer suppression phenotype in *age-1(mg109)*;*pdk-1(mg261) *animals.

### AKT-1 and PDK-1 are interdependent when activated by mutations

**Figure 3:**
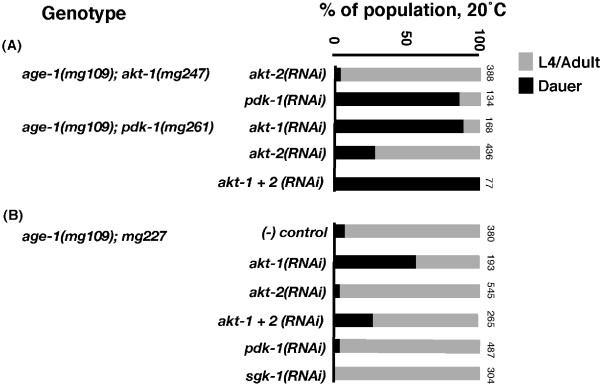
**RNAi epistasis analysis of *age-1(mg109) *suppressors reveals interdependence of AKT-1 and PDK-1**. To establish whether *akt-1(mg247), pdk-1(mg261) *and *mg227 *mutations suppressed dauer arrest through the AGE-1/PI3 kinase pathway we carried out a series of genetic epistasis experiments using RNAi. Bars show the percentage of animals that bypassed dauer arrest, developing into L4 larvae or adults (grey), and those that arrested as dauers (black). Numbers on the side of the bars represent the total number of animals examined. RNAi experiments were diluted with control L4440 RNAi to match the mixed *akt-1 *and *akt-2 *RNAi dose. (A) *age-1(mg109)*;*akt-1(mg247) *animals arrested as dauer larvae on *pdk-1 *RNAi. Similarly, *age-1(mg109);pdk-1(mg261) *animals arrested as dauer larvae on *akt *RNAi. (B) Epistasis analysis on *age-1(mg109);mg227 *animals suggests *mg227 *requires *akt-1 *but not *akt-2, sgk-1 *or *pdk-1 *to bypass dauer larval arrest.

We next examined the epistatic relationships of *akt-1(mg247) *and *pdk-1(mg261) *with other components of the AGE-1/PI3K effector pathway using RNAi. First, we found that *pdk-1(RNAi) *caused constitutive dauer larval arrest in *age-1(mg109);akt-1(mg247) *animals, suggesting that *mg247*-activated AKT-1 required PDK-1-mediated phosphorylation for activity (Figure 3). In contrast, *akt-2(RNAi) *did not have a significant effect on the development of *age-1(mg109);akt-1(mg247) *animals. PDK-1 is believed to be constitutively active under basal conditions [17,18]. Thus, in *age-1(mg109) *animals carrying the *akt-1(mg247) *allele, PDK-1 may be active at low levels.

Next, we observed that reproductive development of *age-1(mg109);pdk-1(mg261) *animals was suppressed by *akt-1(RNAi)*, and this effect was enhanced in combination with *akt-2(RNAi)*, showing that the activated form of PDK-1 remained coupled to the AKT/PKB outputs (Figure 3). Bypass of dauer larval arrest in *age-1(mg109)*;*akt-1(mg247) *and *age-1(mg109)*; *pdk-1(mg261) *animals was unaffected by RNAi-mediated knockdown of *sgk-1*, which encodes another effector of AGE-1/PI3K signaling (data not shown) [8].

We also analyzed the epistatic interactions of *mg227 *with *akt-1*, *akt-2, sgk-1 *and *pdk-1*. In these experiments, *akt-1(RNAi) *partially suppressed bypass of dauer larval arrest in *age-1(mg109)*; *mg227 *animals, indicating that *mg227 *may act upstream of *akt-1*. However, development in *age-1(mg109);mg227 *animals was not affected by *akt-2, sgk-1 *or *pdk-1 *RNAi (Figure 3).

### *age-1(mg109) *dauer arrest suppressors differentially affect lifespan

**Figure 4:**
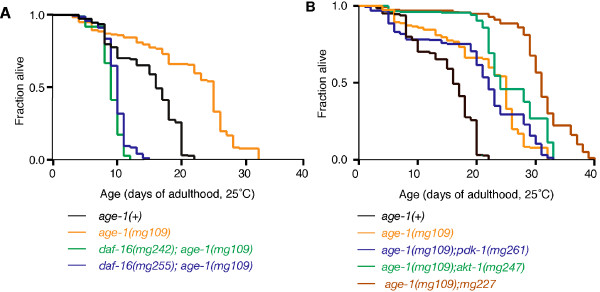
**Lifespan is differentially affected by *age-1(mg109) *dauer arrest suppressors**. Adult lifespan of wildtype, *age-1(mg109) *and suppressor mutations in the *age-1(mg109) *background, as indicated. *age-1(mg109) *animals were maternally rescued *(mg109/mg109*) progeny of *mg109/+ *hermaphrodites. (A) Both *daf-16 *alleles, *mg242 *and *mg255*, suppressed *age-1(mg109) *adult longevity. The *mg242 *allele, which affects both *daf-16 *splice forms, had a slightly stronger effect on *age-1(mg109) *longevity than the *daf-16a*-specific *mg255 *allele (*P *< 0.001, *mg242 *vs.*mg255*). (B) The *akt-1 *(*mg247) *and the *pdk-1 *(*mg261) *alleles did not significantly affect *age-1(mg109) *adult longevity. The *mg227 *allele enhanced *age-1(mg109) *adult longevity. Adult lifespan data is shown in Table 2.

Although all 5 of these alleles could suppress dauer arrest in *age-1(mg109) *animals, there were significant differences in their effects on the *age-1(mg109) *adult longevity and stress resistance phenotypes. Only the *daf-16 *alleles, *mg242 *and *mg255*, fully suppressed *age-1(mg109) *adult longevity (Log-Rank test, *P *≤ 0.0001 vs. *age-1(mg109)*, Table 2; Figure 4A). In addition, *age-1(mg109) *animals carrying either the *mg242 *or *mg255 *alleles also lived significantly shorter than wildtype animals, consistent with previous reports of shortened lifespan by *daf-16 *mutations [16,31]. The *daf-16(mg242) *allele, which disrupts both the *daf-16a *and *daf-16b *isoforms, caused a slightly greater lifespan reduction in *age-1(mg109) *animals, than the *daf-16a*-specific *mg255 *allele (Log-Rank test *P *≤ 0.0001 *mg242 *vs. *mg255*). However, this difference was minor, indicating that the *daf-16a *isoform provides the majority of the DAF-16 function required for longevity in *age-1(mg109) *animals.

**Table 2:** Adult lifespan of *age-1(mg109) *dauer arrest suppressors.

Genotype	Adult Lifespan			
	
	Mean	SE	*(n)*	Log-Rank
Wildtype ^a^	14.9	0.33	255	-
*age-1(mg109) *^a, b^	21	0.66	142	-
*daf-16(mg242);age-1(mg109) *^a^	9.0	0.11	225	<0.0001^g^
*daf-16(mg255);age-1(mg109) *^a^	9.8	0.11	283	<0.0001^g^
*age-1(mg109);akt-1(mg247) *^a^	25.5	0.42	205	<0.0001^c, g^
*age-1(mg109);pdk-1(mg261) *^a^	20.2	0.69	166	<0.6941^d, g^
*age-1(mg109);mg227 *^a^	30.5	0.38	279	<0.0001^g^
				
Wildtype; control RNAi ^f^	22.9	0.49	53	-
Wildtype; *daf-16 *RNAi ^f^	16.4	0.26	107	-
*age-1(mg109);mg227*; *daf-16 *RNAi ^f^	18.7	0.27	97	

^a ^Lifespan assays performed at 25°C

^b ^These are homozygote progeny of *age-1(mg109) *homozygous hermaphrodites that have been maternally rescued for dauer arrest [27].

^c ^Although the *akt-1(mg247) *allele increased mean lifespan compared to *age-1(mg109) *control animals, the median lifespan and maximum lifespan was not significantly affected (*age-1(mg109)*^a, b^, 25 days median, 32 days maximum; *age-1(mg109);akt-1(mg247)*^a^, 24 days median, 33 days maximum, Figure 4)

^d ^Wilcoxan *P *value = 0.2370.

^f ^Lifespan assays performed at 20°C.

^g ^*P *vs *age-1(mg109)*^a, b^

SE, Standard error.

*n *indicates the number of animals scored in at least three independent trials.

Neither *akt-1(mg247) *nor *pdk-1(mg261) *suppressed *age-1(mg109) *adult longevity (Table 2; Figure 4B). Interestingly, the *mg227 *allele enhanced longevity of *age-1(mg109) *adults (Log-Rank test *P *= < 0.0001 vs.*age-1(mg109*), Table 2; Figure 4B) and this enhancement was *daf-16 *dependant (Table 2).

### Dauer arrest suppressors differentially affect stress resistance and FIRE response

**Figure 5:**
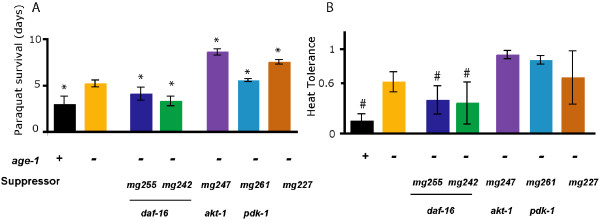
**The *age-1(mg109) *stress resistance phenotype is differentially altered by dauer arrest suppressors**. (A) Oxidative stress was assessed by treatment with 10 mM paraquat on normal agar medium from the first day of adulthood. *age-1(mg109) *animals were more resistant to oxidative stress when compared to wildtype animals. Both *daf-16 *alleles suppressed the *age-1(mg109) *stress resistant phenotype. Neither *akt-1(mg247)*, *pdk-1(mg261) *or *mg227 *suppressed oxidative stress resistance of *age-1(mg109)*. *P < 0.0001, vs. *age-1(mg109)*. Mean survival ± SE, *age-1(+)*, 2.76 ± 0.07 days (*n *= 96); *age-1(mg109)*, 5.14 ± 0.10 days, (*n *= 107); *daf-16(mg255);age-1(mg109)*, 4.02 ± 0.09 days, (*n *= 112); *daf-16(mg242);age-1(mg109)*,3.22 ± 0.05 days, (*n *= 122); *age-1(mg109);akt-1(mg247)*, 8.6 ± 0.18 days, (*n *= 112);*age-1(mg109);pdk-1(mg261)*, 5.96 ± 0.32 days, (*n *= 63);*age-1(mg109);mg227*, 7.48 ± 0.27 days, (*n *= 87). (B) Synchronized animal populations were subjected to heat stress at 35°C for 16 hours and scored for survival. Shown is fraction alive in 3 experiments with 60 – 100 animals/experiment; error bars, SEM, #*P *< 0.05. *age-1(mg109) *animals were more resistant to heat stress when compared to wildtype animals and both *daf-16 *alleles suppressed the *age-1(mg109) *stress resistant phenotype. However, neither *akt-1(mg247)*, *pdk-1(mg261) *or *mg227 *suppressed the stress resistance of *age-1(mg109)*. Mean fraction alive ± SD, *age-1*(+), 0.14 ± 0.14; *age-1(mg109)*, 0.61 ± 0.20; *daf-16(mg255);age-1(mg109)*, 0.39 ± 0.29; *daf-16(mg242);age-1(mg109)*, 0.35 ± 0.43; *age-1(mg109);akt-1(mg247)*, 0.93 ± 0.08; *age-1(mg109); pdk-1(mg261)*, 0.87 ± 0.08; *age-1(mg109);mg227*, 0.66 ± 0.5.

Reductions in AGE-1/PI3K signaling are associated with increased resistance to oxidative and thermal stresses [22,23]. We therefore examined the effects of dauer arrest suppressors on survival of *age-1(mg109) *animals under conditions of oxidative and thermal stress. Both *daf-16 *alleles, *mg242 *and *mg255*, completely suppressed oxidative stress resistance of *age-1(mg109) *animals tested by treatment with 10 mM paraquat, an intracellular free radical generator (Figure 5a). However, neither *akt-1(mg247)*, *pdk-1(mg261) *or *mg227 *suppressed oxidative stress resistance of *age-1(mg109) *animals to paraquat. Double mutant *age-1(mg109) *animals carrying any of these suppressor alleles appeared to have increased survival in the presence of 10 mM paraquat, compared to parental *age-1(mg109) *animals (Figure 5a. Similar results were observed for suppression of thermotolerance of *age-1(mg109) *adults, as tested by survival at the stressful temperature of 35°C (Figure 5b).

**Figure 6:**
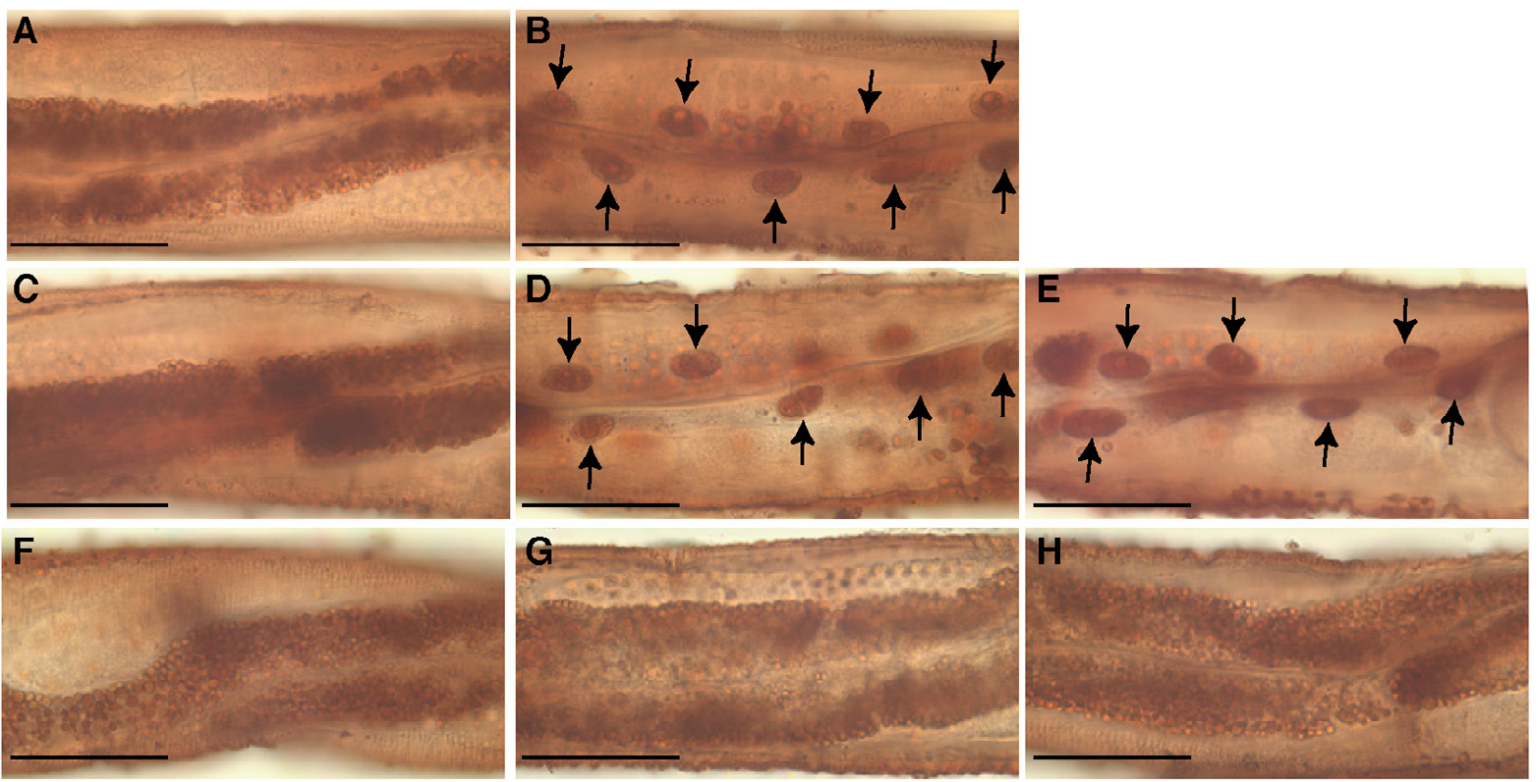
**Suppression of altered FIRE response in *age-1(mg109) *animals**. Distribution of intestinal esterase activity in (A) well-fed or (B) fasted *age-1(+) *young adult animals exhibited the fasting-induced redistribution (arrows) of esterase from the intestinal cytoplasm (A) to a nuclear pattern (B). (C) *age-1(mg109) *adults were resistant to the FIRE response. Altered FIRE response of *age-1(mg109) *animals was suppressed by (D) *daf-16(mg242) *and (E) *daf-16(mg255) *alleles, but not by (F) *akt-1(mg247)*, (G) *pdk-1(mg261) *or (H) *mg227 *mutations. Scale bars, 50 μm.

We also analyzed the intestinal response to fasting in these strains by monitoring the FIRE response (Fasting-Induced Redistribution of Esterase activity) [32]. After a 6-hour fast, intestinal esterase activity in wildtype adults redistributes from cytoplasmic to nuclear localization (Figure 6A, B). This response is altered in *age-1(mg109) *adults, which continue to exhibit the cytoplasmic pattern of esterase activity after a 6-hour fast (Figure 6C). Both the *daf-16(mg242) *and *daf-16(mg255) *mutations suppressed the altered FIRE response in *age-1(mg109) *animals, while *akt-1(mg247)*, *pdk-1(mg261) *and *mg227 *had no effect on the altered FIRE response of *age-1(mg109) *animals (Figure 6D–H).

**Figure 7:**
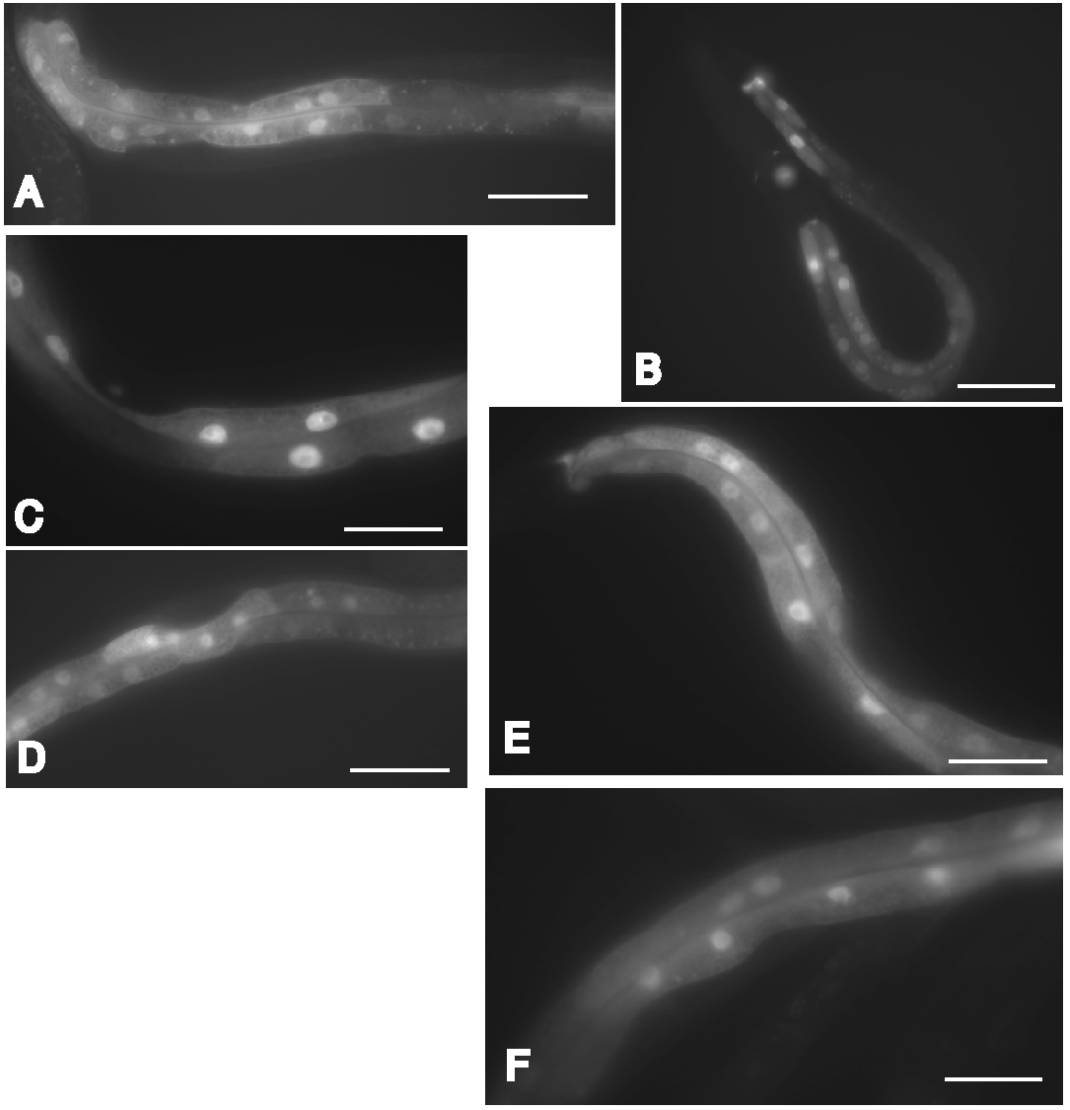
***age-1(mg109) *suppressor mutants differentially redistributes DAF-16:GFP localization**. Intestinal DAF-16:GFP localization redistributed to the nucleus of wildtype animals after a period of heat stress (35°C) (A, B). DAF-16:GFP localization is primarily in the nucleus of *daf-16(mg255);age-1(mg109) *animals (C) reflecting localization of DAF-16 in a *age-1(mg109) *animal. In contrast, DAF-16:GFP localization was seen in both the cytoplasm and nucleus of *age-1(mg109);akt-1(mg247) *animals (D), *age-1(mg109);pdk-1(mg261) *animals (E) and *age-1(mg109);mg227 *(F). Scale bar 50 μm.

Finally, we investigated the effects of these mutations on DAF-16:GFP localization. In wildtype animals, 35°C heat stress could induce the nuclear localization DAF-16:GFP translational fusion (Figure 7A, B). In unstressed wildtype animals DAF-16:GFP was detected in both the nucleus and cytoplasm. Defects in the insulin signaling pathway should cause nuclear accumulation of DAF-16:GFP as previously reported [13-15]. We observed that DAF-16:GFP fluorescence was predominantly nuclear in *age-1(mg109) *adults containing the *daf-16(mg255) *mutation (Figure 7C). In contrast, DAF-16:GFP showed both nuclear and some cytoplasmic localization in *age-1(mg109) *animals carrying the *akt-1(mg247)*, *pdk-1(mg261) *or *mg227 *alleles (Figure 7D–F). Thus we conclude that the mutations activating AKT signaling may partially rescue DAF-16:GFP nuclear localization by the *age-1(mg109) *allele.

In *C. elegans*, signaling by the DAF-2/insulin receptor, via AGE-1/PI3K, regulates developmental arrest at the dauer larval stage, as well as longevity and stress resistance in adult animals. Here, we report the characterization of new mutations identified through a genetic screen designed to identify components of the AGE-1/PI3K signaling pathway. This screen identified 2 mutations inactivating one or two isoforms of *daf-16*, which encodes the FOXO transcription factor that is the ultimate output of AGE-1/PI3K signaling. Both of the *daf-16 *alleles suppressed all the *age-1(mg109) *dauer and adult phenotypes examined, consistent with the conclusion that *daf-16 *activity is necessary for both the larval and adult phenotypes of *age-1 *mutant animals. Additionally, we identified 3 mutations that uncoupled the larval and adult phenotypes of AGE-1/PI3K signaling. These mutations appear to activate the AKT/PKB effector pathway of AGE-1/PI3K signaling, as evidenced by the dependence of these 3 alleles on wildtype *akt-1 *and/or *pdk-1 *activity. Previous reports also identified activating mutations in *akt-1 *and *pdk-1*, yet ours is the first report to probe in depth the effects of this class of mutations on adult AGE-1/PI3K functions [11,12]. In addition, we report the phenotypic characterization of a new mutation, *mg227*, which has the unusual phenotype of enhancing adult longevity in *age-1(mg109) *animals in addition to suppressing larval dauer arrest.

In this work, we identified an activating mutation in the AKT-1 linker region between the PH and kinase domains. Interestingly, the previously identified *mg144 *allele also contained a linker region mutation (Ala183Thr) [12]. The linker region in mammalian AKT/PKB proteins is considerably shorter than in the *C. elegans *protein, so it is not clear whether linker region function is conserved in the *C. elegans *and human proteins or whether the *C. elegans *linker region possesses additional functions. The structure of the PH domain from human AKT has been determined [33]. This AKT fragment contained part of the linker region which appeared to face away from the residues that mediate phospholipid binding and probably does not play a significant role in determining lipid binding specificity. Instead, linker region mutations have been postulated to relax autoinhibition of the kinase and PH domains in the inactive AKT protein [12]. Interestingly, our epistasis analysis showed that the gain-of-function phenotype of *akt-1(mg247) *required the presence of wild type *pdk-1*. This suggests that the mutant protein encoded by *akt-1(mg247) *may be more easily phosphorylated by PDK-1 in the absence of AGE-1/PI3K-generated phospholipids

We also identified an activating allele of *pdk-1*, encoding the kinase that phosphorylates and activates phospholipid-bound AKT/PKB. The *mg261 *mutant contains mutations of both a nonconserved residue in the linker region and a conserved residue in the PH domain which may mediate binding to the phospholipid products of AGE-1/PI3K [34,35]. A previously identified activated *pdk-1 *allele, *mg142*, corresponded to an Ala303Val substitution in the PDK-1 kinase domain and was shown to increase the basal level of PDK-1 kinase activity [11]. The regulation of PDK-1 signaling is complex and depends on the downstream target in question [36]. In *C. elegans*, the major targets of PDK-1 are believed to be AKT-1, -2 and SGK-1 [8,11,12]. One possibility is that the *mg261 *mutation in the PH domain could alter the specificity of PDK-1 phospholipid binding, enhancing PDK-1 activity in the absence of AGE-1/PI3K phospholipid products. However, PDK-1 is active in *age-1(mg109) *as evidenced by *age-1(mg109);akt-1(mg247) *mutants. Therefore it is not entirely clear how the *pdk-1(mg261) *allele is activating AKT-1.

*daf-16 *activity is required for both the larval dauer arrest and adult longevity and stress resistance phenotypes that result from genetic deficiencies in the *age-1 *pathway [21,27]. Consistent with previous findings, the *daf-16 *alleles described here suppressed both larval and adult phenotypes of *age-1(mg109) *animals. In contrast, the *akt-1(mg247)*, *pdk-1(mg261) *and *mg227 *alleles did not affect the *age-1(mg109) *adult phenotypes. Thus, the larval and adult outputs of AGE-1/PI3K signaling are differentially affected by *akt-1(mg247), pdk-1(mg261) *and *mg227 *alleles. It was previously shown that *pdk-1 *activity is necessary for wildtype adult lifespan, demonstrating that PDK-1 mediates the adult outputs of AGE-1/PI3K signaling [11]. Thus, we propose that the differential suppression by *pdk-1(mg261) *of the *age-1(mg109) *larval versus adult phenotypes results from insufficient activation of PDK-1 activity for adult, but not larval, phenotypes.

A similar explanation may account for the inability of *akt-1(mg247) *to suppress adult phenotypes of *age-1(mg109) *animals. However, a previous report suggested that SGK-1, and not AKT-1 or -2, may be the primary mediator of DAF-2 and AGE-1 signaling in adult animals [8]. Thus, it is possible that *akt-1 *itself does not affect adult-specific functions, but only the dauer-specific functions of insulin signaling. However, we have observed that the DAF-16::GFP reporter is distributed in the cytoplasm in adult *age-1(mg109); akt-1(mg247) *animals, suggesting that *mg247 *at least partially restores wildtype regulation of DAF-16 in adult animals. Thus, there may be additional pathways that converge with AGE-1/PI3K signaling to regulate DAF-16 activity and, ultimately, lifespan in adult animals.

**Figure 8:**
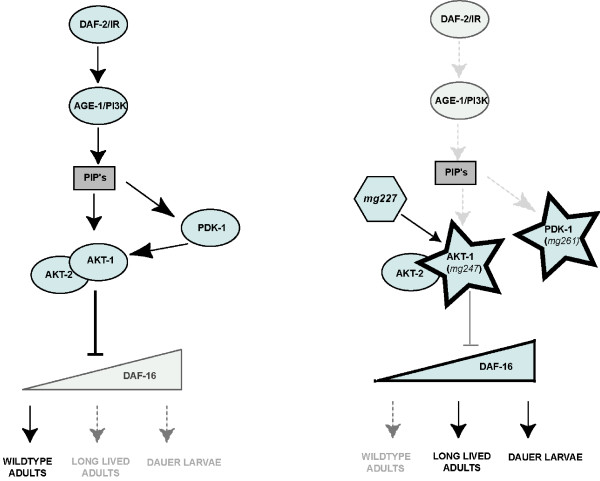
**Model for regulation of insulin pathway phenotypes by AKT-1 and PDK-1 activation**. Activated signaling components in the absence of AKT-1 signaling in *age-1(mg109) *mutant background. See text for details.

One possible explanation for the different effects of these mutations on the larval versus adult phenotypes of *age-1(mg109) *animals is that they partially restore insulin pathway signaling and negative regulation of DAF-16. According to this model, wildtype levels of insulin pathway signaling may strongly inhibit DAF-16 and promote reproductive development and adult lifespan. Whereas, low insulin signaling promotes both dauer larval arrest and adult longevity [19-21]. The mutations activating AKT signaling appear to promote intermediate levels of insulin pathway signaling, allowing reproductive development, but prolonging adult lifespan. This phenotypic gradient could reflect a gradient of DAF-16 activity in these strains, in which the highest levels of DAF-16 activity causes dauer larval arrest (Figure 8). Thus the mutations activating AKT signaling may allow an intermediate level of DAF-16 activity, which is insufficient to promote dauer larval arrest, but does promote adult longevity. This is similar to a DAF-16 gradient model that has been previously proposed [8].

## Conclusion

In this study we identified mutations capable of activating signaling in the absence of AGE-1/PI3K. We describe five mutations including two mutations inactivating one or two isoforms *daf-16 *and two new hypermorphic alleles of *akt-1 *and *pdk-1*. The *daf-16 *alleles described in this work suppress *age-1(mg109) *dauer and adult phenotypes examined consistent with previous reports. The *akt-1 *and *pdk-1 *alleles uncouple the dauer arrest, adult longevity and stress resistance phenotypes of *age-1(mg109) *mutants. Furthermore, genetic epistasis analysis suggests these hypermorphic alleles are interdependent. These findings suggest larval and adult phenotypes of insulin signaling are fully separable in these mutants.

## Methods

### Strains and general methods

The *C. elegans *strains used in this work were N2 Bristol (wildtype), *sqt-1(sc13) age-1(mg109)/mnC1*; and CB4856 Hawaiian. Strains were provided by the *Caenorhabditis *Genetics Center at the University of Minnesota. All strains were cultivated at 15°C on NGM agar plates seeded with the *E. coli *strain OP50 following standard protocols [37].

### Suppressor isolation and mapping

Suppressors were isolated as previously described on a parent strain carrying the *sqt-1(sc13) age-1(mg109) *mutation balanced with *mnC1 *and F_2 _progeny were screened for suppression of the *age-1(mg109) *constitutive dauer arrest phenotype [12]. From a screen of 20,000 haploid genomes, 40 suppressors were identified of which 5 were crossed with Hawaiian strain males (CB4856). DNA from these suppressor recombinants were collected and analyzed using SNP-snip mapping for each chromosome [29]. For candidate gene sequencing, genomic DNA from the mutant strains (*sqt-1(sc13) age-1(mg109); m/m*) was PCR amplified with exon-specific primers and sequenced to identify molecular changes. *mg227 *SNP-snip mapping showed strong linkage to the right arm of chromosome X between 10.5 – 13.5 m. u. Candidate genes were PCR amplified and sequenced. Genes sequenced included known dauer forming genes, *daf-12*, (F11A1.3, X2.39), *lin-14 *(T25C12.1, X4.28), *bra-1 *(F54B11.6, X13.84), *akt-2 *(F28H6.1, X16.56) and *sgk-1 *(W10G6.2, X2.70). Furthermore, a cosmid carrying the *daf-12 *gene did not rescue the *mg227 *daf-C suppression phenotype in *age-1(mg109);mg227 *animals (5 trangenic lines examined, 94 % of animals bypassed dauer arrest, 6 % arrested as dead eggs).

### RNA interference

A 500 bp region spanning the largest exon in both *akt-2 *and *pdk-1 *was amplified from N2 DNA and cloned into the RNAi feeding vector L4440 [38]. *akt-1 (RNAi) *and *daf-16 (RNAi) *was obtained from the RNAi feeding library [30]. LB broth supplemented with 100 μg/mL of ampicillin was inoculated with RNAi clones and grown overnight at 37°C without shaking. RNAi experiments were typically carried out on 6-well plate NGM media supplemented with 50 μg/mL ampicillin and 1 mM IPTG. Overnight bacterial cultures were seeded on duplicate wells and allowed to grow at room temperature for a further 12 – 16 hr. G_1 _and G_2 _progeny of fertile hermaphrodites were collected in a 5-hour egg lay on RNAi bacteria. The G_2 _progeny were allowed to grow at 20°C for 3 days after which any phenotypes were scored. Plates were re-examined 24 hr later for changes in phenotypes if any. Each experiment was done in triplicate.

### Phenotypic assays

To assay dauer larval arrest, gravid adult hermaphrodites were allowed to lay eggs at room temperature for 4 hours to produce a synchronized population. Animals were then shifted to 25°C or 20°C and left to develop for a further 72 or 96 hours after which the number of dauers and adults (sterile or fertile) was counted. Sterility was scored as the absence of embryos in the uterus in young adults (day 1–2).

For lifespan analysis, larvae were raised at 15°C until the first day of adulthood (Day 0) prior to being shifted to 25°C on standard NGM plates supplemented with FUDR (50 μg/mL). Survival analyses were always performed in the presence of FUDR, since some of these strains have an egg-laying defect that causes eggs to accumulate within the uterus and hatch, thereupon killing the parent hermaphrodite. FUDR treatment helps to remedy this problem as it is an inhibitor of DNA synthesis and prevents embryonic development. Animals were scored every two days for death by failure to respond to gentle prodding on the head and tail. All aging experiments were repeated in at least three separate trials and cumulative results were analyzed. Lifespan statistical analysis was performed using the JMP software package (version 5.1).

For paraquat and thermotolerance survival, young adults were obtained as for lifespan assays. For paraquat treatments, day 0 adults were shifted to standard NGM plates supplemented with 10 mM paraquat at 20°C. Survival of animals was scored at 2 or 3 day intervals by failure to respond to gentle prodding. For thermotolerance assays, synchronized young gravid adults were placed on bacteria-free NGM plates pre-heated to 35°C. All plates were wrapped in Parafilm to prevent dessication and placed in a 35°C incubator for 16 hours. Survival was scored by failure to respond to gentle prodding after 16 hours.

FIRE response was assayed by scoring cytoplasmic versus nuclear intestinal esterase activity, as described previously [32]. Briefly, stress due to food deprivation was achieved by transferring bacteria-free young adult animals to NGM agar with 100 μg/mL ampicillin and incubating at 25°C for 6 hours. Fasted adults were fixed with methanol (-20°C) for 10 minutes and incubated overnight at room temperature in staining solution described previously [32]. Stained specimens were mounted on 2 % agarose pads and viewed on a Nikon E800 microscope with Nomarski optics. Images were collected using a Hamamatsu ORCA-ER CCD camera using OpenLab software (Improvision, Lexington, MA).

### DAF-16:GFP localization

A *gfpdaf-16 *cDNA fusion was constructed using an intestinal *gly-19 *promoter as previously described [32]. In brief, the DAF-16:GFP plasmid was constructed by inserting a *gfp *coding sequence in-frame with the *daf-16a *full-length cDNA. The intestinal promoter was cloned upstream of *gfpdaf-16 *using unique Sph*I *and Kpn*I *sites. Plasmid were injected into adult hermaphrodites at a final concentration of 25–100 μg/mL [32].

## Authors' contributions

MSG carried out genetic mapping of *daf-16(mg242), daf-16(mg255), akt-1(mg247) *and *mg227*, lifespan analysis, RNAi experiments, oxidative stress and heat stress assays on all mutations, and wrote the manuscript. WBI performed the FIRE assays and DAF-16:GFP localization analysis. KH carried out mapping of *pdk-1(mg261)*. CAW carried out the genetic screen as a postdoctoral fellow in Gary Ruvkun's laboratory and wrote the manuscript.
